# Scientometrics analysis of research activity and collaboration patterns in Chagas cardiomyopathy

**DOI:** 10.1371/journal.pntd.0006602

**Published:** 2018-06-18

**Authors:** Gregorio González-Alcaide, Alejandro Salinas, José M. Ramos

**Affiliations:** 1 Department of History of Science and Documentation, University of Valencia, Valencia, Spain; 2 Service of Internal Medicine, Hospital de Denia, Alicante, Spain; 3 Department of Clinical Medicine, Miguel Hernández University of Elche, Alicante, Spain; 4 Department of Internal Medicine, Hospital General Universitario de Alicante, Alicante, Spain; Instituto de Ciências Biológicas, Universidade Federal de Minas Gerais, BRAZIL

## Abstract

**Background:**

Chagas cardiomyopathy is a serious and common complication of Chagas disease.

**Methods:**

Through bibliometric and Social Network Analysis, we examined patterns of research on Chagas cardiomyopathy, identifying the main countries, authors, research clusters, and topics addressed; and measuring the contribution of different countries.

**Results:**

We found 1932 documents on Chagas cardiomyopathy in the MEDLINE database. The most common document type was ‘journal article’, accounting for 79.6% of the total (n = 1538), followed by ‘review’ (n = 217, 11.2%). The number of published records increased from 156 in 1980–1984 to 311 in 2010–2014. Only 2.5% were clinical trials. Brazil and the USA dominated the research, participating in 53.1% and 25.7%, respectively, of the documents. Other Latin American countries where Chagas is endemic contributed less, with Bolivia, where Chagas disease is most prevalent, producing only 1.8% of the papers. We observed a high rate of domestic collaboration (83.1% of the documents published in 2010–2016) and a lower but significant rate of international collaboration (32.5% in the same time period). Although clinical research dominated overall, the USA, Mexico and several countries in Europe produced a considerable body of basic research on animal models. We identified four main research clusters, focused on heart failure and dysfunction (physical symptoms, imaging techniques, treatment), and on myocarditis and parasitemia in animal models.

**Conclusions:**

Research on Chagas cardiomyopathy increased over the study period. There were more clinical than basic studies, though very few of the documents were clinical trials. Brazil and the USA are currently leading the research on this subject, while some highly endemic countries, such as Bolivia, have contributed very little. Different approaches could help to redress this imbalance: encouraging researchers to conduct more clinical trials, launching international collaborations to help endemic countries contribute more, and strengthening links between basic and clinical research.

## Author summary

Scientific production on Chagas cardiomyopathy has grown considerably since the turn of the 21st century, probably reflecting the increased incidence of Chagas disease in non-endemic areas like the USA and Europe. Brazil and the USA dominate the research, but we found a very small proportion of clinical trials on Chagas cardiomyopathy and a low scientific production in several endemic countries with a high prevalence of the disease such as Colombia, Chile, Mexico and Bolivia. We observed a polarity between endemic and non-endemic countries where clinical research and basic research predominate, respectively. Different approaches could help to redress the observed imbalance of research on Chagas cardiomyopathy: encouraging researchers to conduct more clinical trials, launching international collaborations to help endemic countries contribute more, and strengthening links between basic and clinical research. It is crucial to foster translational research in order to link basic knowledge on the physiology of the disease with clinical applications in diagnosis and treatment.

## Introduction

Chagas disease, or American trypanosomiasis, is a systemic chronic infection caused by the parasite *Trypanosoma cruzi* and mainly transmitted to humans by reduviid insects. It occurs primarily in Central and South America, affecting an estimated 7.7 million people [1–4]. However, globalization has entailed large-scale population movements, including migratory flows from Latin America to Europe, North America, and elsewhere, and there are now cases reported worldwide [5–6], making the disease a global public health problem.

Chagas disease has two phases: acute and chronic. The acute infection normally manifests as a self-limiting fever. In the chronic phase, around one third of sufferers will develop cardiac or digestive complications within three decades of the initial infection. Some 20% to 30% of people infected with *Trypanosoma cruzi* develop Chagas cardiomyopathy [7–10], a complication with no characteristic signs or symptoms to distinguish it from general heart disease [11]. According to the estimates based on 2010 data, the number of cases of Chagas cardiomyopathy in countries of Latin America stands at 1.17 million people (1,171,193). Estimated numbers were highest in Argentina (376,309), Brazil (231,364), Colombia (131,388) and Bolivia (121,437), followed by Mexico (70,117) [12]. Currently, doctors still rely on nonspecific criteria for its diagnosis, namely a cardiothoracic ratio greater than 50% or abnormalities detected by electrocardiography or echocardiography [11, 13]. While not exclusive to Chagas cardiomyopathy, right bundle branch block and arrhythmias might be considered distinguishing features more commonly observed than in non-Chagas cardiomyopathy. For diagnosis of Chagas cardiomyopathy, the key points are that infection would first be diagnosed via serology, and there are currently no known biomarkers to reliably predict which T. cruzi positive patients will progress to Chagas cardiomyopathy, although research is underway [7, 14]. A new screening method based on brain natriuretic peptide levels and diastolic function could detect early cardiac involvement in Chagas disease [15], potentially overcoming the limitations of traditional diagnostic methods that may misclassify patients. Such advances are highly significant, as the condition is the most common non-ischemic cardiomyopathy and a leading cause of complications and death in Latin America [16]. It carries a risk of malignant arrhythmias, conduction disturbances, heart failure, and pulmonary and systemic embolism, killing around 4% of patients treated for the condition on an outpatient basis every year.

Given the disease burden associated with Chagas cardiomyopathy, a specific analysis of research publications and collaboration networks in this area is warranted to build on the more general bibliometric studies of Chagas disease [17–18]. Increased knowledge on research in this pathology can help to foster North-South collaborations and other research initiatives with and among endemic countries that nevertheless may have relatively little scientific development on the topic. Moreover, this type of study is useful for the research community, clarifying the main lines of research that are being developed with regard to the diagnostic methods and treatments for the disease.

In this study, by analyzing scientific papers on Chagas cardiomyopathy published in the main international scientific journals, we aimed to identify the leading researchers, the contribution of different countries to the overall research effort, the degree and nature of scientific collaboration, and the topics addressed.

## Methods

This study is based on bibliometric methods and Social Network Analysis, through which we collected relevant information from the scientific records indexed in the main bibliographic databases of scientific literature.

### Identifying the population of documents

We selected the body of documents for our study from the MEDLINE and Science Citation Index Expanded (SCI-Expanded) databases. MEDLINE is the main international database for health sciences, and the terminology included in its Medical Subject Headings (MeSH) thesaurus can be used to search for published documents on specific aspects of a given topic. For our search, we identified all the documents indexed in the MEDLINE database with the MeSH descriptor of “Chagas cardiomyopathy”. We then restricted the results to the “article” and “review” document types and to the 1980–2016 period for the calculation of all indicators and analyses, as articles and reviews are the main document types of reference that are subjected to peer review with regard to the dissemination of research activities.

The use of the MeSH thesaurus ensures that all the documents recovered focus on the topic analyzed, as this detailed instrument for controlling terminology combines the use of a team of specially trained indexers who analyze each article and assign medical subject headings to it with automated functions to improve the indexing process [19–21]. To identify the documents with a primary focus on Chagas cardiomyopathy, we screened search results by hand, analyzing titles, abstracts and key words. Moreover, we classified all documents that used “humans” or “humans” and “animals” as clinical, epidemiological, or basic studies.

One limitation of MEDLINE is that until 2013, journal articles included only the address of the first author. To depict more precisely the geographical distribution of research activities and collaboration, we identified the documents from MEDLINE that were also included in SCI-Expanded (61.64% of the MEDLINE documents), as this database shows the addresses of all authors. Moreover, as SCI-Expanded is a multidisciplinary database containing the publications with the greatest visibility and impact, it can reveal the patterns of collaboration in the most internationally relevant journals and enables the analysis of the impact of the publications, based on the citations they generate.

We performed the electronic searches on 25 March 2017 using the platform Web of Science, which contains both databases. Although we did not limit our results by date of publication, it is worth noting that the term “Chagas cardiomyopathy” was not in the MeSH thesaurus until 1981.

### Indicators obtained and aspects analysed

#### Scientific activity and collaboration

We examined the evolution in the number of records published over the years as well as their distribution by document type and journal. We also analysed the geographical distribution of scientific production by institutions and by countries, and the extent and nature of scientific collaboration. We identified, in that sense, the documents with no collaboration (signed only by authors from a single institution) along with the documents arising from domestic (authors affiliated with at least two national institutions) and international collaboration (with authors from at least two countries participating). We created a graphic network to visually depict the collaboration between countries, with the thickness of the links representing the strength of collaboration (number of documents published together). Additionally, we determined the country of the first and the corresponding author of each of the documents, estimating the relative contribution of each country based on these indicators, which are frequently associated with a larger role or a higher degree of research participation and leadership.

#### Characteristics of main research clusters and leading researchers

We identified the most productive authors and research clusters in the field, which we represented graphically within a co-authorship network. A research cluster can be defined as a group of investigators with a high density of interconnections, affording certain homogeneity and distinguishing them from other clusters.

To determine the most productive authors’ influence in the network, we calculated their betweenness, a centrality measure widely used in Social Network Analysis to measure the degree to which a node (in this case, an author) enables connections between other nodes. We used Pajek program to calculate this indicator.

#### Topics addressed

To identify the specific topics addressed by researchers, we measured the frequency of MeSH used and analysed their content. We also created a co-occurrence network to identify firstly the groupings and interrelationships of the descriptors, and secondly the connections between the descriptors and the previously identified research clusters. We excluded the most generic terms (Chagas cardiomyopahy, *Trypanosoma cruzi*, Chagas disease, humans and animals) from the analysis to focus on the more specific descriptors.

We created the collaboration networks at country and co-author level using the Pajek program, and the MeSH co-occurrence network using VOSviewer.

#### Citation analysis

In order to determine the research impact in the area, we have calculated the total number of citations, the mean citations per document and the H index of the most productive institutions and author clusters identified.

## Results

### Document types and evolution of number of documents published

We retrieved 1932 papers from the MEDLINE database for the whole study period. The most common document type was ‘journal article’, accounting for 79.6% of the total (n = 1538), followed by ‘review’ (n = 217, 11.2%). Other prominent document types that we identified were ‘letter’ (n = 127, 6.6%) and ‘editorial’ (n = 43, 2.2%). We also identified document types by clinical interest. In that sense, only 2.5% of the published documents were clinical trials, and 9.7% were case reports; other document types appear only occasionally (Table 1).

**Table 1:** Distribution of the document types by clinical interest, as assigned under the Chagas cardiomyopathy descriptor in MEDLINE.

Document type	N	%
Case Reports	188	9.7
Clinical Trial	49	2.5
Evaluation Studies	13	0.7
Meta-Analysis	5	0.3
Observational Study	4	0.2
Practice Guideline	3	0.1
Validation Studies	1	0.0

**Fig 1:**
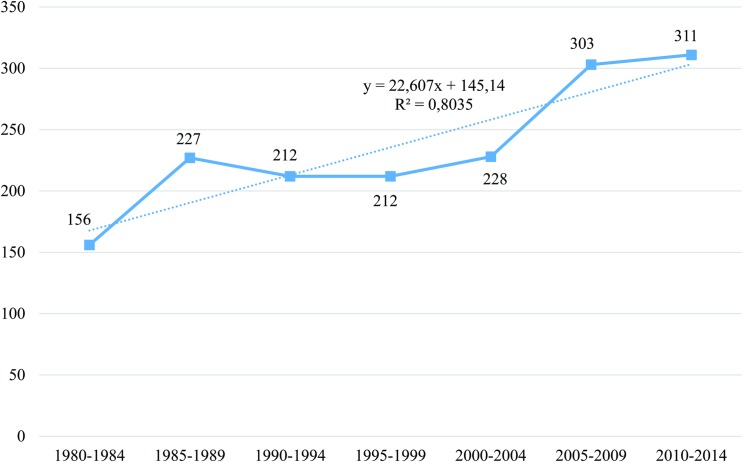
Evolution of number of documents on Chagas cardiomyopathy in the MEDLINE database*. *In the biennium 2015–2016, n = 76.

Fig 1 shows the number of articles and reviews (n = 1755) in MEDLINE on Chagas cardiomyopathy by five-year periods. From 1980–1984 to 1985–1989 there was a 45.5% increase in the number of publications. The number of documents remained stable for the next three five-year periods, before increasing again by 32.9% from 2000–2004 to 2005–2009, then by 2.6% from 2005–2009 to 2010–2014. When the number of publications was plotted over time, the best fit to the data was a straight line (coefficient of determination for linear model, r^2^ = 0.803).

### Journal of publication

The records were published in 382 scientific journals. Four journals contained 25.2% of the Chagas cardiomyopathy literature. About half the literature was concentrated in 20 journals, while the remaining half was scattered throughout the other 362; 221 journals featured only one paper on the topic. Table 2 lists the 24 journals containing the most records, showing their impact factor, subject category and ranking in the 2015 Journal Citation Reports (JCR) as well as their country and language of publication. Two of these journals were not included in the JCR because they had no impact factor. The most common subject categories among the core journals (>14 papers) were “Cardiac & Cardiovascular systems” (n = 7), “Tropical Medicine” (n = 7), “Parasitology” (n = 4), and “Medicine, general & internal” (n = 3). Nine journals were published in the USA, nine in Latin America (six in Brazil plus one each in Argentina, Mexico, and Chile) and six in Europe (one each in France, Germany, Ireland, the Netherlands, Spain, and the UK).

**Table 2:** The 24 journals publishing the most reports on Chagas cardiomyopathy, their impact factor, subject category and ranking in the 2015 *Journal Citation Reports*; and their country and language of publication.

Journal	No. art.	%*	Accumulated %	Impact factor 2015	Journal category (ranking)	Country	Language*
*Arquivos Brasileiros de Cardiologia*	220	12.5	12.5	1.194	Cardiac & cardiovascular systems (97/124) (Q4)	Brazil	Por
*Revista da Sociedade Brasileira de Medicina Tropical*	90	5.1	17.7	0.949	Parasitology (29/36) (Q4) Tropical Medicine (12/19) (Q3)	Brazil	Eng
*Memorias do Instituto Oswaldo Cruz*	68	3.9	21.5	1.789	Parasitology (18/36) (Q2)) Tropical Medicine (6/19) (Q2)	Brazil	Eng
*International Journal of Cardiology*	64	4.6	25.2	4.638	Cardiac & cardiovascular systems (20/124) (Q4)	Ireland	Eng
*The American Journal of Tropical Medicine and Hygiene*	61	3.5	28.7	2.453	Public, Environmental & Occupational Health (47/173) (Q2) Tropical Medicine (4/19) (Q1)	USA	Eng
*Medicina (Buenos Aires)*	42	2.4	31.0	0.589	Medicine, General & Internal (116/155) (Q4)	Argentina	Spa
*Revista do Instituto de Medicina Tropical de Sao Paulo*	33	1.9	32.9	1.114	Tropical Medicine (10/19) (Q2)	Brazil	Mul
*Plos Neglected Tropical Diseases*	31	1.8	34.7	3.949	Parasitology (6/36) (Q1) Tropical Medicine (1/19) (Q1)	USA	Eng
*Archivos del Instituto de Cardiologia de Mexico*†	31	1.8	36.5	—	—	Mexico	Spa
*Brazilian Journal of Medical and Biological Research*	28	1.6	38.1	-‡	- ‡	Brazil	Spa
*American Heart Journal*	26	1.5	39.5	4.332	Cardiac & cardiovascular systems (26/124) (Q4)	USA	Eng
*Circulation*	26	1.5	41.0	17.202	Cardiac & cardiovascular systems (2/124) (Q4) Peripheral vascular disease (1/63) (Q1)	USA	Eng
*Transactions of The Royal Society of Tropical Medicine and Hygiene*	24	1.4	42.4	1.631	Public, Environmental & occupational Health (87/173) (Q3) Tropical Medicine (7/19) (Q2)	UK	Eng
*Sao Paulo Medical Journal*	21	1.2	43.6	0.955	Medicine, General & Internal (95/155) (Q3)	Brazil	Eng
*Infection and Immunity*	20	1.1	44.7	3.603	Immunology (56/151) (Q2) Infectious Diseases (28/83) (Q1)	USA	Eng
*Pacing and Clinical Electrophysiology*: *PACE*	19	1.1	45.8	1.440	Cardiac & cardiovascular systems (88/124) (Q3) Engineering, Biomedical (47/76) (Q3)	USA	Eng
*The American Journal of Cardiology*	18	1.0	46.8	3.154	Cardiac & cardiovascular systems (43/124) (Q4)	USA	Eng
*Microbes and Infection*	17	1.0	47.8	2.291	Immunology (102/151) (Q3) Infectious Diseases (46/83) (Q3) Microbiology (70/123) (Q3)	France	Eng
*Parasitology Research*	16	0.9	48.7	2.27	Parasitology (15/36) (Q2)	Germany	Eng
*Revista Medica de Chile*	16	0.9	49.6	0.40	Medicine, General & Internal (1/155) (Q1)	Chile	Spa
*Acta Tropica*	16	0.9	50.5	2.380	Parasitology (12/36) (Q2) Tropical Medicine (5/19) (Q2)	Netherlands	Eng
*Plos One*	15	0.8	51.4	3.057	Multidisciplinary Sciences (11/63) (Q1)	USA	Eng
*Revista Española de Cardiologia*	15	0.8	52.2	4.596	Cardiac & cardiovascular systems (22/124) (Q4)	Spanish	Spa
*Journal of Infectious Diseases*	15	0.8	53.1	6.44	Immunology (20/151) (Q1) Infectious Diseases (5/83) (Q1) Microbiology (14/123) (Q1)	USA	Eng

Por: Portuguese; Eng: English; Spa: Spanish; Mul: Multi-language.

*Percentage of total records found in MEDLINE

†From 2001: *Archivos de Cardiología de México*, SCImago Journal Rank (SJR), Cardiology and Cardiovascular Medicine: 0.15 (255/340) (Q4).

‡SCImago Journal Rank (SJR), Medicine (miscellaneous): 0.545 (593/1826) (Q2).

### Geographic distribution of research

The 1070 documents recovered from SCI-Expanded were published by authors from 35 countries. Table 3 presents data concerning the production of papers from the most productive institutions (>19 documents) and Table 4, the scientific production in each country.

**Table 3:** Citation indicators of the most productive institutions on Chagas cardiomyopathy.

Institution	N docs.	Total citations	Average citation per document ±SD	H Index
Universidade de São Paulo (Brazil)	202	6297	31.17 (59.8)	41
Fundação Oswaldo Cruz (FIOCRUZ) (Brazil)	146	3951	27.06 (34.9)	34
Universidade Federal de Minas Gerais (Brazil)	118	2841	24.08 (26.2)	31
Universidade Federal do Rio de Janeiro (Brazil)	44	1023	23.25 (25.3)	19
Universidad de los Andes (Venezuela)	32	659	20.59 (22.8)	15
Universidad de Buenos Aires (Argentina)	29	716	24.69 (29.5)	14
Albert Einstein College of Medicine (USA)	29	581	20.03 (19.9)	13
Universidade Federal do Triângulo Mineiro (Brazil)	26	793	30.50 (53)	13
Universidade Federal de Ouro Preto (Brazil)	23	228	9.91 (8.9)	10
Universidade Federal de São Paulo (Brazil)	23	378	16.43 (24.4)	11
Consejo Nacional de Investigaciones Científicas y Técnicas (CONICET) (Argentina)	23	697	30.30 (29.1)	14
Universidad de Chile (Chile)	23	279	12.13 (16.5)	9
Universidad Nacional de Córdoba (Argentina)	22	313	14.23 (12.3)	11
University of Texas Medical Branch (USA)	20	502	25.10 (22.9)	12

**Table 4:** Distribution by country of Chagas cardiomyopathy records indexed in SCI-Expanded, and number and % of documents containing the MeSH descriptors “humans” and “animals”.

Country	Total docs		“Humans”		“Animals”		“Humans” and “animals”		Clinical		Epidemiological		Basic research	
	N	%*	N	%†	N	%†	N	%†	N	%	N	%	N	%
Argentina	173	16.2	74	42.8	40	23.1	59	34.1	47	27.2	18	10.4	108	62.4
Australia	1	0.1	1	100	0	0	0	0	0	0	0	0	1	100
Belgium	9	0.8	2	22.2	4	44.4	3	33.3	3	33.3	0	0	6	66.7
Bolivia	19	1.8	14	73.7	0	0	5	26.3	11	57.9	4	21	4	21
Brazil	568	53.1	314	55.3	138	24.3	116	20.4	214	37.7	56	9.9	298	52.5
Canada	17	1.6	12	70.6	3	17.6	2	11.8	7	41.2	3	17.6	7	41.2
Chile	28	2.6	19	67.9	1	3.6	8	28.6	11	39.3	11	39.3	6	21.4
Colombia	44	4.1	30	68.2	2	4.5	12	27.3	16	36.36	4	9.09	24	54.55
Cuba	1	0.1	1	100	0	0	0	0	1	100	0	0	0	0
Czech Republic	2	0.2	0	0	2	100	0	0	0	0	0	0	2	100
Ecuador	3	0.3	3	100	0	0	0	0	2	66.7	1	33.3	0	0
El Salvador	2	0.2	2	100	0	0	0	0	1	50	1	50	0	0
Finland	1	0.1	1	100	0	0	0	0	1	100	0	0	0	0
France	44	4.1	14	31.8	13	29.5	17	38.6	5	11.4	5	11.4	34	77.3
Germany	17	1.6	8	47.1	3	17.6	6	35.3	5	29.4	2	11.8	10	58.8
Guatemala	2	0.2	1	50	0	0	1	50	0	0	1	50	1	50
Israel	3	0.3	1	33.3	0	0	2	66.7	1	33.3	0	0	2	66.7
Italy	20	1.9	16	80	2	10	2	10	13	65	3	15	4	20
Japan	9	0.8	4	44.4	2	22.2	3	33.3	4	44.4	1	11.1	4	44.4
Mexico	27	2.5	8	29.6	5	18.5	14	51.9	6	22.2	8	29.6	13	48.1
Netherlands	1	0.1	1	100	0	0	0	0	1	100	0	0	0	0
Norway	1	0.1	1	100	0	0	0	0	0	0	0	0	1	100
Panama	1	0.1	1	100	0	0	0	0	1	100	0	0	0	0
Paraguay	3	0.3	0	0	2	66.7	1	33.3	0	0	1	33.3	2	66.7
Peoples R China	3	0.3	0	0	1	33.3	2	66.7	1	33.3	0	0	2	66.7
Peru	4	0.4	2	50	1	25	1	25	2	50	0	0	2	50
Singapore	1	0.1	0	0	1	100	0	0	0	0	0	0	1	100
South Africa	1	0.1	0	0	1	100	0	0	0	0	0	0	1	100
Spain	33	3.1	19	57.6	4	12.1	10	30.3	9	27.3	5	15.1	19	57.6
Sweden	1	0.1	0	0	0	0	1	100	0	0	0	0	1	100
Switzerland	10	0.9	3	30	2	20	5	50	4	40	4	40	2	20
UK	19	1.8	9	47.4	3	15.8	7	36.8	6	31.6	4	21	9	47.4
Uruguay	1	0.1	0	0	1	100	0	0	0	0	0	0	1	100
USA	275	25.7	108	39.3	93	33.8	74	26.9	75	27.3	32	11.6	168	61.1
Venezuela	77	7.2	44	57.1	9	11.7	24	31.2	31	40.3	8	10.4	38	49.3

*Percentage of total records.

† Percentage of total documents published in the country

Authors from Latin America and the Caribbean produced by far the most reports on Chagas cardiomyopathy (82.9%). North America ranked second (26.8%) and Europe, third (13.6%). Brazil was the most productive country (53.1%), followed by the USA (25.7%)—where Chagas is not endemic—and Argentina (16.2%). The next most productive endemic countries were Venezuela (7.2%), Colombia (4.1%), Chile (2.6%), Mexico (2.5%), and Bolivia (1.8%); while the next most productive non-endemic countries were France (4.1%), Spain (3.1%), Italy (1.9%), the UK (1.8%), Germany (1.6%), and Switzerland (0.9%) (Table 4).

### Collaboration

Of the 1070 documents found in SCI-Expanded, 736 (68.8%) involved domestic collaborations between different departments or institutions in a single country. International collaborations produced 278 (26%) of the documents. Table 5 shows the evolution of domestic and international collaboration from 1980 to 2016. Domestic collaborations were almost three times as prevalent as international collaborations, though both types increased progressively over the study period.

**Table 5:** Domestic and international collaboration on Chagas cardiomyopathy, records indexed in SCI-Expanded.

Period	No. docs	Domestic collaboration		International collaboration	
		n	%	n	%
1980–1989	136	65	47.8	26	19.1
1990–1999	208	112	53.8	53	25.5
2000–2009	405	293	72.3	95	23.4
2010–2016	320	266	83.1	104	32.5
1980–2016	1070	736	68.8	278	26.0

Most countries in Latin America and the United States show a high degree of domestic collaboration (54%–66% of the documents in which they participated), with the exception of Bolivia (26.3%) and Mexico (44.4%). More significant are the differences observed in relation to international collaboration, with countries like Chile (21.4%), Brazil (29.7%) and Argentina (36.4%) showing values that are far below those observed in the United States and especially European countries (61%–100%). On the other hand, Bolivia (94.7%) and Colombia (75%) do present high degrees of international collaboration (Table 6).

**Table 6:** Analysis of collaboration and leadership at country level in research production on Chagas cardiomyopathy.

Country	Total docs	%	No collaboration	%	Domestic collaboration	%	International collaboration	%	1st position in documents in International collaboration	%	Corresponding (all documents)	% of total scientific production in the country
Argentina	173	16.2	110	63.6	93	53.8	63	36.4	30	47.6	118	68.2
Australia	1	0.1	1	100	0	0	0	0	0	0	1	100
Belgium	9	0.8	2	22.2	5	55.5	7	77.8	1	14.3	4	44.4
Bolivia	19	1.8	1	5.3	5	26.3	18	94.7	3	16.7	2	10.5
Brazil	568	53.1	399	70.2	376	66.2	169	29.7	101	59.8	442	77.8
Canada	17	1.6	0	0	2	11.8	17	100	6	35.3	6	35.3
Chile	28	2.6	22	78.6	17	60.7	6	21.4	2	33.3	21	75
Colombia	44	4.1	11	25	25	56.8	33	75	15	45.4	25	56.8
Cuba	1	0.1	1	100	1	100	0	0	0	0	1	100
Czech Republic	2	0.2	0	0	0	0	2	100	0	0	0	0
Ecuador	3	0.3	0	0	0	0	3	100	1	33.3	1	33.3
El Salvador	2	0.2	1	50	1	50	1	50	0	0	1	50
Finland	1	0.1	0	0	0	0	1	100	0	0	0	0
France	44	4.1	10	22.7	17	38.6	34	77.3	9	26.5	15	34.1
Germany	17	1.6	5	29.4	8	47.1	12	70.6	7	58.3	11	64.7
Guatemala	2	0.2	0	0	0	0	2	100	0	0	0	0
Israel	3	0.3	3	100	3	100	0	0	0	0	3	100
Italy	20	1.9	1	5	4	20	19	95	6	31.6	5	25
Japan	9	0.8	3	33.3	6	66.7	6	66.7	2	33.3	3	33.3
Mexico	27	2.5	14	51.8	12	44.4	13	48.1	6	46.1	15	55.6
Netherlands	1	0.1	0	0	0	0	1	100	0	0	0	0
Norway	1	0.1	0	0	1	100	1	100	0	0	0	0
Panama	1	0.1	1	100	1	100	0	0	0	0	1	100
Paraguay	3	0.3	0	0	2	66.7	3	100	0	0	0	0
Peoples R China	3	0.3	1	33.3	1	33.3	2	66.7	0	0	1	33.3
Peru	4	0.4	0	0	2	50	4	100	1	25	1	25
Singapore	1	0.1	1	100	0	0	0	0	0	0	1	100
South Africa	1	0.1	0	0	0	0	1	100	0	0	0	0
Spain	33	3.1	13	39.4	17	51.5	20	60.6	10	50	24	72.7
Sweden	1	0.1	1	100	1	100	0	0	0	0	0	0
Switzerland	10	0.9	5	50	3	30	5	50	0	0	3	30
UK	19	1.8	5	26.3	2	10.5	14	73.7	2	14.3	3	15.8
Uruguay	1	0.1	1	100	1	100	0	0	0	0	1	100
United States	275	25.7	125	45.4	158	57.4	150	54.5	66	44	162	58.9
Venezuela	77	7.2	55	71.4	42	54.5	22	28.6	10	45.4	43	55.8

Brazil is the country that stands out the most in terms of leading collaborative papers, as authors of this country occupy the position of first author in 59.8% of the collaborative publications in which Brazilian authors participated, and of corresponding author in 77.8%. These values are markedly higher than those seen in the United States (44% as first authors and 58.9% as corresponding authors). Germany and Spain also hold positions of leadership on these indicators, while Bolivia is at the bottom of the ranking on both (Table 6).

**Fig 2:**
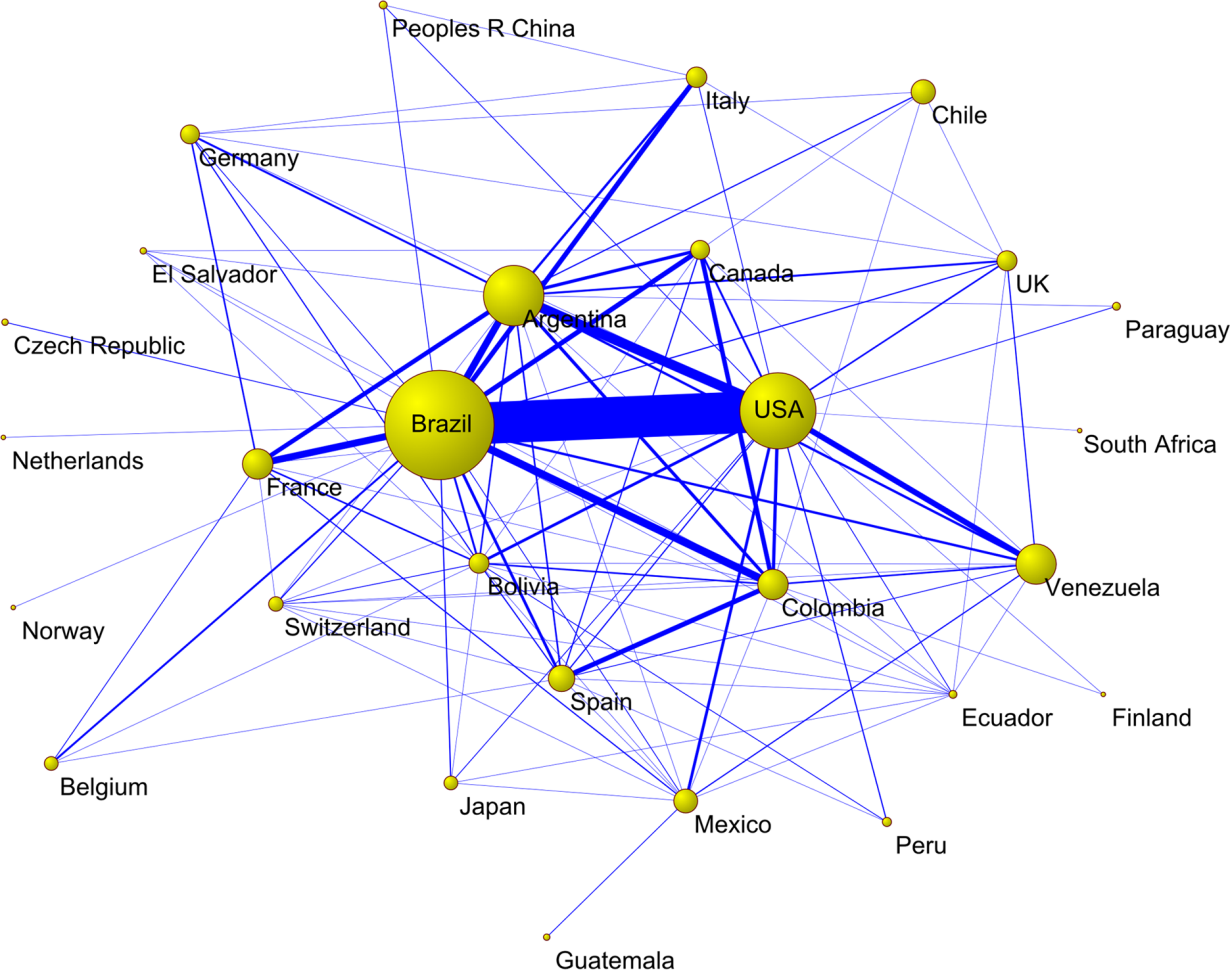
Collaboration network between countries on Chagas cardiomyopathy, records indexed in SCI-Expanded.

Fig 2 shows the collaboration network between countries. By far the strongest cooperative link was between Brazil and the USA, with 102 documents published in collaboration. The USA also had notable links with other Latin American countries such as Argentina (n = 24) and Venezuela (n = 14); Brazil had important links with other Latin American countries such as Colombia (n = 19) and Argentina (n = 17), and European countries such as France (n = 17) and Italy (n = 12).

### Authorship and research clusters

The 1755 papers (articles and reviews) in MEDLINE were produced by 4686 authors responsible for 9858 signatures. The average number of authors per paper over the whole study period was 5.6. This number was less than 2.5 in the first period analysed (1949 to 1979), 4.6 in 1980–1989, 4.8 in 1990–1999, 5.9 in 2000–2009 and 7.4 in 2010–2016.

**Fig 3:**
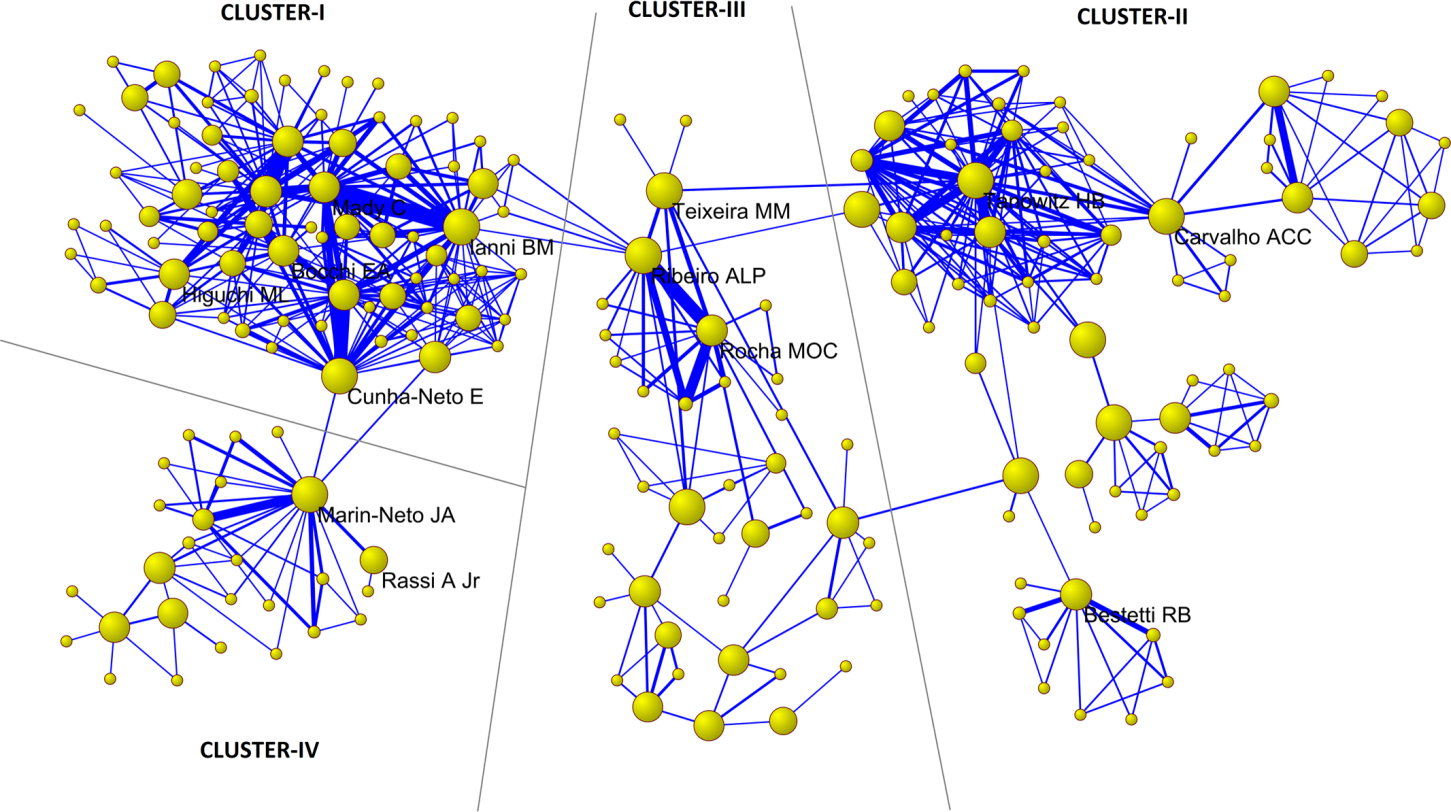
Main research foci of the co-authorship network, generated from the documents on Chagas cardiomyopathy indexed in MEDLINE.

Fig 3 depicts the main research foci of the co-authorship network, comprising 207 authors who are directly or indirectly interlinked and who have co-authored four or more documents together. We identified four research clusters: cluster I contained the largest number of authors (n = 72), of whom B. M. Ianni and E. Cunha-Neto had established the most links; in cluster II (n = 69) the most prominent author was H. B. Tanowitz; in cluster III (n = 40), A. L. P. Ribeiro; and in cluster IV (n = 26), J. A. Marin-Neto.

Table 7 shows the ranking of the 30 most productive authors (≥ 24 papers) and the authors with the highest values of betweenness centrality in the co-authorship network. The most productive author was C. Mady (n = 65), followed by M. O. C. Rocha (n = 58), and F. Pileggi (n = 57). The most influential cutpoints in the networks were J. A. Marin-Neto, followed by A. C. C. Carvalho and A. L. P. Ribeiro. Thirteen of the 30 authors appear in both lists (Fig 3).

**Table 7:** Most productive authors (>50 records) and authors with highest values of betweenness centrality in Chagas cardiomyopathy papers indexed in MEDLINE.

Productivity			Betweenness centrality		
Rank	Author	N docs	Rank	Author	Value
1	Mady C	65	1	Marin-Neto JA	0.086922
2	Rocha MOC	58	2	Carvalho ACC	0.064900
3	Pileggi F	57	3	Ribeiro ALP	0.061380
4	Ianni BM	53	4	Higuchi ML	0.049663
4	Marin-Neto JA	53	5	Garg NJ	0.041857
6	Ribeiro ALP	51	6	Cunha-Neto E	0.035044
7	Tanowitz HB	50	7	Mady C	0.034097
8	Bellotti G	48	8	Xavier SS	0.031581
9	Bestetti RB	47	9	Teixeira MM	0.029785
9	Cunha-Neto E	47	10	Rassi A Jr	0.029649
11	Kalil J	39	11	Araujo-Jorge TC	0.027069
12	Bocchi EA	38	12	Sosa EA	0.025525
13	Rossi MA	37	13	Silva JS	0.025439
14	Stolf NAG	36	14	Guzman-Bracho C	0.025424
15	Higuchi ML	35	15	Chiale PA	0.024764
16	Carvalho ACC	34	16	Tanowitz HB	0.024711
17	Nunes MCP	33	17	Morillo CA	0.023711
18	Rassi A Jr	32	18	Sgammini H	0.023431
19	Teixeira MM	30	19	Rocha MOC	0.023047
20	Reyes PA	29	20	Ianni BM	0.022476
21	Andrade ZA	27	21	Elizari MV	0.022334
22	Andrade SG	26	22	Bocchi EA	0.022315
22	Carrasco HA	26	23	Paola AAV de	0.022050
22	dos Santos RR	26	24	Diez C	0.021792
25	Barretto ACP	25	25	Bestetti RB	0.021572
25	Davila DF	25	26	Grecco OT	0.021507
25	Jelicks LA	25	27	Correa-Oliveira R	0.021383
25	Maciel BC	25	28	Lopes ER	0.021306
29	Soares MBP	24	29	Dias JCP	0.020487
30	Weiss LM	24	30	Bottasso OA	0.019793

### Classification of the type of research study and analysis of MeSH

The manual revision of the studies we retrieved showed that 1225 documents (69.8%) mentioned Chagas cardiomyopathy in the title or abstract, indicating that this was the central focus of the publications. There were also 336 documents (19.1%) that referred to Chagas disease and 176 (10%) to *Trypanosoma cruzi* in the title or abstract. While these studies focused on those subject areas, the abstracts also mentioned terms like cardiomyopathy, myositis, heart, or another word that justified their inclusion under the MeSH descriptor of Chagas cardiomyopathy. Finally, 18 documents (1%) include a generic description of cardiovascular disease in Latin America.

Table 8 lists the 100 most frequently used MeSH in documents on Chagas cardiomyopathy. The term “humans” (n = 1412) appeared twice more often than “animals” (n = 722). We assigned the “humans” descriptor to 58.9% (n = 1033) of the documents and the “animals” descriptor to 19.4% (n = 341), while we used both descriptors for 21.7% (n = 381). Of the documents reporting studies on humans, 63.9% (n = 660) were clinical studies that addressed aspects related to diagnosis or therapeutic strategies; 16.1% (n = 166) were epidemiological studies, and 20% (n = 207) reported basic research on immunological, biochemical or molecular aspects of the disease. The documents classified under the joint “human-animal” descriptor were also most commonly rooted in basic research approaches (43.3%, n = 165), while the rest were on animal models (17.6%, n = 67), clinical research studies (24.9%, n = 95) or epidemiological studies (14.2% n = 54). Overall, 43% of the documents were clinical (n = 755), 12.5% (n = 220) epidemiological, and 44.4% either animal (n = 408) or basic (n = 372) studies.

**Table 8:** Top 100 Medical Subject Headings (MeSH) from Chagas cardiomyopathy, records indexed in MEDLINE.

MeSH	N docs	%	MeSH	N docs	%
Chagas Cardiomyopathy	1755	100	Prospective Studies	57	3.2
Humans	1412	80.4	Cytokines	56	3.2
Male	986	56.2	Disease Progression	56	3.2
Female	867	49.4	Polymerase Chain Reaction	56	3.2
Middle Aged	740	42.2	Biomarkers	55	3.1
Animals	722	41.1	Dogs	53	3.0
Adult	697	39.7	Young Adult	52	3.0
Trypanosoma cruzi	576	32.8	Heart Block	49	2.8
Chagas Disease	444	25.3	Cells, Cultured	46	2.6
Chronic Disease	420	23.9	Heart Conduction System	46	2.6
Electrocardiography	341	19.4	Age Factors	45	2.6
Aged	339	19.3	Hemodynamics	45	2.6
Myocardium	324	18.5	Tachycardia, Ventricular	45	2.6
Mice	287	16.3	Blood Pressure	44	2.5
Adolescent	180	10.2	Prevalence	44	2.5
Heart Failure	168	9.6	Child, Preschool	42	2.4
Heart	165	9.4	Cross-Sectional Studies	42	2.4
Disease Models, Animal	146	8.3	Fibrosis	41	2.3
Myocarditis	136	7.7	Severity of Illness Index	41	2.3
Antibodies, Protozoan	124	7.1	Exercise Test	40	2.3
Brazil	121	6.9	Recurrence	40	2.3
Arrhythmias, Cardiac	115	6.5	United States	40	2.3
Echocardiography	114	6.5	Bundle-Branch Block	39	2.2
Heart Ventricles	106	6.0	Myocytes, Cardiac	39	2.2
Acute Disease	105	6.0	Genetic Predisposition to Disease	37	2.1
Prognosis	100	5.7	Interferon-gamma	37	2.1
Heart Rate	99	5.6	Autonomic Nervous System	36	2.0
Rats	99	5.6	Genotype	36	2.0
Follow-Up Studies	98	5.6	Immunohistochemistry	36	2.0
Child	95	5.4	T-Lymphocytes	36	2.0
Cardiomyopathy, Dilated	87	4.9	Cardiomyopathies	35	2.0
Time Factors	76	4.3	Infant	35	2.0
Risk Factors	75	4.3	Magnetic Resonance Imaging	35	2.0
Case-Control Studies	74	4.2	Molecular Sequence Data	35	2.0
Myocardial Contraction	73	4.1	Survival Analysis	35	2.0
Trypanocidal Agents	70	4.0	Tumor Necrosis Factor-alpha	35	2.0
Parasitemia	69	3.9	CD8-Positive T-Lymphocytes	34	1.9
Antigens, Protozoan	67	3.8	Death, Sudden, Cardiac	33	1.9
Treatment Outcome	67	3.8	Inflammation	33	1.8
Ventricular Dysfunction, Left	67	3.8	Diagnosis, Differential	32	1.8
Heart Transplantation	66	3.8	Ventricular Function, Left	32	1.8
Mice, Inbred C57BL	63	3.6	Defibrillators, Implantable	31	1.8
Immunoglobulin G	63	3.6	DNA, Protozoan	31	1.8
Enzyme-Linked Immunosorbent Assay	60	3.4	Host-Parasite Interactions	31	1.8
Autoantibodies	60	3.4	Mice, Inbred C3H	31	1.8
Nitroimidazoles	60	3.4	Sensitivity and Specificity	31	1.8
Stroke Volume	59	3.4	Survival Rate	31	1.8
Aged, 80 and over	58	3.3	Autoimmunity	30	1.7
Mice, Inbred BALB C	58	3.3	Mexico	30	1.7
Retrospective Studies	58	3.3	Pacemaker, Artificial	30	1.7

The most commonly used MeSH related to human research were “Chronic Disease” (n = 420), “Electrocardiography” (n = 341), “Heart Failure” (n = 168), “Arrhythmias, cardiac” (n = 115), “Echocardiography” (n = 114), and other descriptors related to patient follow-up and prognosis assessment. Some descriptors, such as “Myocarditis” (n = 136) and “Parasitemia” (n = 69) were more common in animal-based research.

Brazil (n = 121) is the main geographic MeSH term assigned to the documents, followed at considerable distance by the United States (n = 40), Mexico (n = 30), Chile (n = 23), Argentina (n = 19) and Bolivia (n = 16).

**Fig 4:**
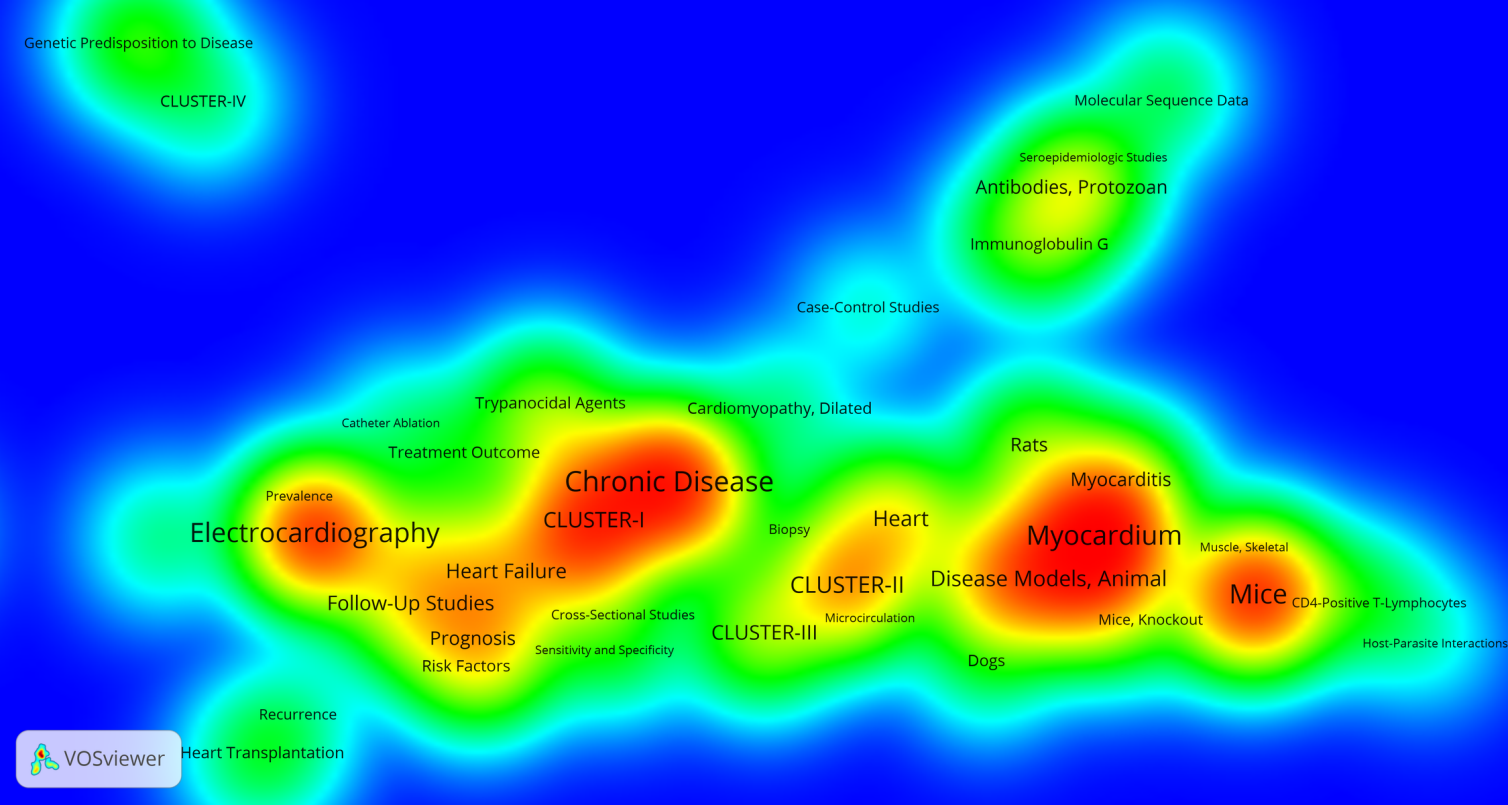
Network of MeSH and their association with the research clusters.

Fig 4 maps the most common MeSH, showing how they are linked to the four research clusters. There are two main areas of research: heart failure and dysfunction (MeSH describing physical symptoms, graphic representation techniques, treatments and outcomes), shown on the left of the graph; and animal models on the right. The figure reveals the thematic focus of each of the four research clusters. Cluster I has a strong clinical focus, whereas cluster II is more closely associated with research on animal models. Cluster III is the most heterogeneous of the four, with ties to both approaches. For its part, cluster IV is associated with the study of genetic aspects of the disease.

When we analysed the documents by country of publication, we found some striking differences in research foci (Table 4). Human-based research is most prominent in Brazil, with 55.3% of records containing the MeSH “humans”, versus 24.3% for “animals”. This trend was even more pronounced in Bolivia (73.7% of records containing “humans”), Chile (67.9%), Colombia (68.2%) and Venezuela (57.1%). In the USA, although human-based research predominated, a considerable proportion of records were related to animals (39.3% vs 33.8%, respectively). We found a similar trend in France (31.8% vs 29.5%) and Mexico (29.6% vs 18.5%). The distribution of the documents by study type confirms the predominance of the clinical approach in countries like Bolivia (57.9% of the documents) or Chile (39.3%, compared to 21.4% of documents reporting basic research). It also reflects the hegemony of the basic approach in the United States (61.1%), European countries like France (77.3%) and Spain (57.9%), and in Argentina (62.4%) and Mexico (48.1%).

### Research impact

The most productive institutions generally present a higher degree of citation, with the Universidade de São Paulo the main reference center with regard to all citation indicators. The Fundação Oswaldo Cruz (FIOCRUZ) and the Universidade Federal de Minas Gerais also stand out in terms of the absolute number of citations received and the H index. Other prominent institutions include the Universidade Federal do Triângulo Mineiro, the Consejo Nacional de Investigaciones Científicas y Técnicas (CONICET) and the University of Texas Medical Branch, which all present a high average number of citations per document despite showing a more moderate absolute production (Table 3).

With regard to the clusters of authors and their citations, cluster II shows the highest absolute degree of citation (n = 5336) and the highest H index (n = 37), with an average number of citations per document of 26.8 (SD ±34). Cluster IV, despite its lower degree of citation (n = 3167) and H index (n = 28), the average number of citations per document is greater (41.7 ±90.4). Documents published by authors of cluster I yielded a total of 3932 citations (mean 33.6 SD ±43.8; H index = 35), while publications by authors from cluster III generated 2937 (mean 23.3 SD ±25.8; H index = 32). All of the clusters present mean degrees of citation above the average observed for the whole set of documents analyzed (23 SD ±36.3), with cluster III showing the lowest levels of citation among the research groups, despite its relevance as a bridge between the other clusters. This relatively moderate impact may be a reflection of the lower number of authors and their disperse relationships, which show a lower density of research ties.

## Discussion

### Scientific activity and international collaboration

Scientific production on Chagas cardiomyopathy has grown considerably since the turn of the 21st century, probably reflecting the increased incidence of Chagas disease in non-endemic areas like the USA and Europe, and the possibility of providing new knowledge of the disease, with the introduction of treatment projects and new diagnostic tests. Another relevant factor is the increase in domestic and international collaboration, which stimulates further research [15, 22–24].

Our findings revealed a high degree of domestic collaboration, especially in recent years, in response to the multidisciplinary nature of this clinical entity, which is relevant to tropical medicine, parasitology, cardiology, pathology, biochemistry and immunology, infectious diseases, physiology, microbiology and public health [25–26].

We found that most research on Chagas myocardiopathy was concentrated in journals related to cardiovascular systems, tropical medicine and parasitology, which is logical because the disease affects the heart, is caused by a parasite, and is most prevalent in tropical and subtropical regions. Brazil and other South American countries, together with the USA, produced the most papers on Chagas cardiomyopathy, for two main reasons: firstly, Chagas disease mainly affects Latin American populations; and secondly, the USA, a leader in the research of this subject, has established many collaborative ties with these countries, especially with Brazil [17–18]. Among the published documents, 9.7% were case studies and only 2.5% clinical trials, probably because measuring the efficacy of the few existing therapeutic tools is highly problematic [25, 27]. Thus, pharmacological treatments and antiarrhythmic therapies for Chagas myocardiopathy are often based on results for other etiologies. Specific clinical trials are crucial for helping health professionals to better understand and manage the complication [1].

Our findings showed Brazil at the center of the collaboration network, with strong links to other countries in Central and South America (particularly the bordering Argentina, Venezuela and Colombia) and also to non-endemic countries—primarily the USA, but also some countries in Europe. The fact that Brazil and the United States are the two primary geographic MeSH terms assigned to the documents confirms the research leadership exercised by these countries, but it also reflects the high prevalence of the disease in Brazil and the fact that the USA also has vectors and documented transmission [28].

Brazil plays a central role in research on Chagas myocardiopathy because it has the largest population of all Latin American countries and also a strong research sponsorship scheme that has driven international collaboration [29]. Bolivia, on the other hand, has the highest prevalence of Chagas disease and therefore of Chagas myocardiopathy, but it produced only 1.8% of the records in our study, a reflection of its low overall health system expenditure. Strengthening cooperative networks could help to address this deficit. Accordingly, in recent years Bolivia has increased collaborative research with other countries in Latin America, North America, Europe, and even Japan [23, 30].

### Authorship and research clusters

We found that the average number of authors included in the published documents increased over the study period, consistent with all health sciences disciplines. This increase in scientific cooperation will doubtless have several positive consequences, and may help to integrate less developed countries into research activities, but the trend may also be associated with negative aspects that should be avoided wherever possible: processes of neocolonialism or scientific dependence, whereby the more developed countries decide on the lines and topics of research without taking into account the expectations and interests of the countries where the disease has the largest impact [31–32]. The medium-term goal of establishing collaborative links between countries with more and less economic and scientific development should be to empower these latter countries with mutually beneficial and balanced partnerships [33–34].

Among the authors who have established strong collaborative links, we found a few key players who occupy a prominent position, collaborating with several other investigators and acting as intermediaries between different research clusters. These investigators play a crucial role in driving research in the field, since they help to integrate new researchers and generate resources. Moreover, they produce a high volume of work, disseminate information and new ideas, and foster the application of research methodologies and exchange of resources [35–36].

### Research topics

The fact that MEDLINE indexers use the most specific term available in the MeSH branched hierarchy [19] explains the high percentage (70%) of documents tagged as focusing on Chagas cardiomyopathy. This is a specific descriptor for Chagas disease that is only assigned to documents when the title, abstract or key words make explicit reference to it, or when the thematic focus can be deduced by statements linking Chagas or *Trypanosoma cruzi* with a cardiovascular concept.

The proportion of human-based research varied from 55% in Brazil to 74% in Bolivia. The predominance of human-based research in Latin American countries like Bolivia, Brazil, Chile, Colombia and Venezuela reflects the importance of applied clinical practice in Chagas disease in these settings. In contrast, higher income countries without vector-borne transmission of *Trypanosoma cruzi* conduct a higher proportion of animal research. The USA and France are the leaders in this respect, with 34% and 29.5% of papers in these countries containing the MeSH term “animals”, which is roughly balanced with the 39% and 32% of reports related to “humans”. Among the countries in Latin America with the highest scientific production, Brazil (24.3%), Argentina (23.1%) and Mexico (18.5%) conducted the highest proportion of research on animal models. Since the physiopathology of myocardial damage is poorly understood in Chagas disease, animal models are crucial for improving scientific knowledge, and high-income countries are largely the ones generating this research. Thus, their collaboration with countries where the disease is endemic in humans is crucial. Initiatives like the Special Programme for Research and Training in Tropical Diseases (TDR), an independent global program of scientific collaboration cosponsored by the United Nations Children's Fund, the United Nations Development Program, the World Bank, and the World Health Organization, could serve as a model in this respect, as it enables researchers from different countries and institutions to work together on projects related to tropical diseases [25, 37]. The manual revision also showed the relevance of animal research and experimentation in the field of Chagas cardiomyopathy due to the gaps in knowledge around the etiopathogenesis of the *Trypanosoma cruzi* infection and the resulting myocardial damage. Indeed, analysis of the kind of animal experimentation described in the documents that also report basic research in humans shows that nearly half of the research papers on Chagas cardiomyopathy are focused on clarifying the underlying mechanisms of myocardial damage and the factors involved in this pathology.

The most commonly used MeSH related to research in humans described diagnostic imaging tests. This reflects the fact that Chagas cardiomyopathy is a chronic disease that can present as arrhythmia and even heart failure, two complications where traditional and simple diagnostic techniques such as electrocardiography are being displaced by other methods like echocardiography [38–39]. We also found MeSH terms detailing the follow-up of patients with cardiomyopathy, and the evolution of Chagas disease towards cardiomyopathy, which shows that these topics are still poorly understood [25, 40–42]. With regard to animal models, the related MeSH terms reflect an interest in understanding the immunoallergic and microbiological implications of Chagas disease and Chagas myocardiopathy [26, 43].

### Limitations

The main limitation of our study is that the databases used (MEDLINE and SCI-Expanded) do not index some of the journals published in Central and South America, particularly in some countries where the disease is endemic, such as Bolivia, Paraguay and El Salvador. This may have limited the visibility of the papers produced in these countries. We performed the analysis based on searches of MEDLINE and WoS because these are the databases of reference at a bibliometric level and with regard to the most widely disseminated literature at an international level. They also enable far more exact analyses because bibliographic data are standardized, citations of publications are recorded, and search terminology is controlled very precisely through the MeSH.

Furthermore, it is possible that our search strategy, which was based on a clinical manifestation of the disease, could have resulted in a relatively modest presence of basic studies, which may be indexed under “Chagas disease” rather than “Chagas cardiomyopathy”.

Finally, it is worth noting that the use of addresses may not reflect the actual nationality of authors and the geographic setting where the studies took place, due to researcher mobility, the existence of research stays in other countries, and the development of research projects in endemic, low- and middle-income countries that are nevertheless funded and directed by institutions and researchers from high-income countries. Thus, the indicators obtained from authors’ institutional affiliations should be interpreted only as an approximation to the institutions that are responsible for driving the performance of the studies and publishing their results.

### Conclusions

In our study we identified the authors who comprise the main international research foci on Chagas cardiomyopathy, measured the extent of collaboration between authors, and identified the topics covered. It is noteworthy that despite the important degree of research development described, less than 1% of patients are able to access treatment. This wide gap points to the need to translate research results into comprehensive public health policy that extends access to clinical services for the disease. Related to this point, the low number of studies with Bolivian authors and researchers from other countries with a high prevalence of the disease, such as Mexico or Colombia, reflects the concentration of resources and higher education institutions in larger, wealthier countries. There is thus an acute need for capacity building in research infrastructure in endemic countries, especially the development of clinical trials with funding from states, industry and civil society, among other initiatives [44].

Domestic and international collaboration plays an important role in the research on this area. Further international cooperation could help to reduce the concentration of research activities in countries with more developed scientific systems such as Brazil or the USA, and reduce the polarity observed between endemic and non-endemic countries, where clinical research and basic research predominate, respectively. It is crucial to strengthen the link between basic research, which is focused on understanding the physiology of the disease, and clinical research, which concerns diagnosis and treatment.
